# Understanding self-managing teams in Dutch healthcare: empirical evidence to non-sequential team development processes

**DOI:** 10.1108/JHOM-04-2020-0122

**Published:** 2021-03-23

**Authors:** Iris A.G.M. Geerts, Joyce J.P.A. Bierbooms, Stefan W.M.G. Cloudt

**Affiliations:** School of Governance, Faculty of Law, Economics and Governance, Utrecht University , Utrecht, The Netherlands; Research Group Evidence Based Management of Innovation, Geestelijke Gezondheidszorg Eindhoven en De Kempen , Eindhoven, The Netherlands; Tranzo, Tilburg School of Social and Behavioral Sciences , Tilburg University , Tilburg, The Netherlands; Department of Organization Studies, Tilburg School of Social and Behavioral Sciences, Tilburg University , Tilburg, The Netherlands

**Keywords:** Self-managing teams, Team development, Non-sequential model, Team processes, Healthcare

## Abstract

**Purpose:**

This two-part study aims to contribute to the body of knowledge on team development by examining the development of self-managing teams (SMTs) in healthcare. Based on an exploration of the team development literature, a perspective on SMT development was created, which suggested that SMTs develop along a non-sequential pattern of three processes–team management, task management and boundary management and improvement–that is largely the result of individual, team, organizational and environmental-level factors.

**Design/methodology/approach:**

The perspective on SMT development was assessed in a Dutch mental healthcare organization by conducting 13 observations of primary mental healthcare SMTs as well as 14 retrospective interviews with the self-management process facilitator and advisors of all 100 primary mental healthcare SMTs.

**Findings:**

Empirical results supported the perspective on SMT development. SMTs were found to develop along each of the three defined processes in a variety or possible patterns or simultaneously over time, depending on many of the identified factors and three others. These factors included individual human capital, team member attitudes and perceived workload at the individual level, psychological safety, team turnover, team size, nature of the task and bureaucratic history at the team level, and management style and material and social support at the organizational level.

**Practical implications:**

This study provides a non-sequential model of SMT development in healthcare, which healthcare providers could use to understand and foster SMTs development. To foster SMT development, it is suggested that cultural change need to be secured alongside with structural change.

**Originality/value:**

Even though various team development models have been described in the literature, this study is the first to indicate how SMTs in the healthcare context develop toward effective functioning.

## 1. Introduction

Nowadays, healthcare organizations are searching for more cost-efficient ways of delivering care as the current economy asks for reduction of costs, while patients demand a higher quality and more diverse and flexible care. To adequately deal with these demands, healthcare organizations have placed more reliance on organizational flexibility and respond by decentralization. That is, attributing responsibility and autonomy to the workforce by organizing employees into self-managing teams (SMTs) ( Yeatts *et al.* , 2004 ; Renkema *et al.* , 2018 ). An SMT is a permanent group of “interdependent individuals that can self-regulate their behavior on relatively whole tasks” ( Cohen *et al.* , 1996 , p. 644). They share the responsibility for making a product or service and have authority over decisions such as task assignments, working methods and scheduling of activities ( Cohen *et al.* , 1996 ). The rationale for adopting SMTs in healthcare derives from the proposition that they are more effective at allocating their resources, and therefore more flexible in adapting structures to a variety of tasks, situations and conditions in comparison with traditional, hierarchical teams ( Wageman, 1997 ).

In order to harness the flexibility benefits of SMTs, it is crucial to understand how team members develop over time into an effective functioning SMT. One main line of thinking of several theoretical models is that the development pattern of (self-managing) teams can be described as an ordered set of distinct linear phases. In the early phases of development, team members are confronted with interpersonal conflicts, communication blocks, and poor tasks and process skills. Only after some time, if team members move through a number of subsequent phases, do teams reach the point of effective functioning and become completely self-managed ( Tuckman, 1965 ; Van Amelsvoort and Benders, 1996 ).

Even though various other team development models have been proposed in the literature as well, most models seem to have limited relevance for SMTs in healthcare organizations. First, there is limited empirical research focusing on SMT development in particular. This holds both for quantitative and qualitative research. Most studies have focused more on theoretical descriptive models, rather than real, in-context empirical data and, if they are, they tend to be focused on self-analytic groups, (student) project teams or common hierarchical teams ( Kuipers and Stoker, 2009 ; Benders *et al.* , 2014 ). Second, the limited amount of empirical research that did focus on SMT development occurred exclusively within production and manufacturing organizations (i.e. Wellins *et al.* , 1991 ; De Leede and Stoker, 1996 ; Kuipers and De Witte, 2005 ; Kuipers and Stoker, 2009 ; Powell and Pazos, 2017 ). The findings of these studies may not be the same as those for SMTs in healthcare organizations because teams in healthcare work in a complex environment and with a high degree of accountability ( Baker *et al.* , 2006 ), while teams in production and manufacturing are more focused on improving processes and maintaining standards ( Bhuiyan *et al.* , 2006 ). Third, most existing team development models often treat (self-managing) teams as closed systems, thereby ignoring the context in which the team operates and the characteristics of the team that might influence team development ( Gersick, 1988 ; Wheelan, 2005 ; Peralta *et al.* , 2018 ).

Giving the growing prevalence of SMTs in healthcare organizations, a better understanding of SMTs' development pattern and its influencing factors is needed. The present study therefore aims to contribute to the body of knowledge on team development by examining the development of SMTs in healthcare. To do so, this study is divided in two major parts: the first part of this study provides a theoretical exploration of the team development literature to arrive at a perspective on SMT development in healthcare. Subsequently, the second part of this study assesses the perspective on SMT development in a Dutch mental healthcare organization. By examining SMT development in healthcare, insights are provided into how managers, advisors and policymakers can support SMTs in moving further along their development toward effective functioning.

## 2. Theory

In this section, a theoretical exploration of several models, theories and literature on team development is provided to arrive at a perspective on SMT development in healthcare. The search for relevant scientific literature was conducted via the ISI/Web of Science and Google Scholar databases in May 2016. The literature search included a number of different keyword combinations of “team development” OR “group development” AND commonly used concepts of SMTs such as “self-directed team,” “semi-autonomous team” OR “self-organizing team.” To evaluate the relevance of the database hits (over 35.000), the article title and/or information within the abstract was screened. Articles were selected when they were published in 1990 or later (an exception was made for certain “classic works”), fitted in the management, business and social science studies and identified at least two dimensions of change to ensure that articles limited to only one dimension were ruled out (e.g. longitudinal study on conflict). This eventually led to the selection of 20 articles. The literature search was repeated in February 2020 to update the sample with three more recent articles.

### 2.1 Categorization of team development models

Smith's (2001) categorization of linear-progressive, cyclical (or pendular) and non-sequential models was used as a starting point for analyzing the various selected team development models and theories. The reasons for using the framework of Smith (2001) is that it is based on five other fundamental classification frameworks in team development literature and that it allows to classify several of the more recent team development models as well. Hence, Smith's (2001) framework captured all different team development approaches found in the literature.

The framework of Smith (2001) posits that team development models can be classified into one of three categories: linear-progressive models, cyclical (or pendular) models and non-sequential models. The linear-progressive models assume that team development is based “on the completion of a definite and ordered set of stages, phases or periods” ( Smith, 2001 , p. 19). The models included in this category are some of the most-widely cited and have their origins in various approaches such as group-dynamics literature, socio-technical principles or the consultancy practice. Cyclical models are based on the notion that teams revisit different stages or phases, depending on the issues that surface a given time. Each cycle serves to strengthen the team's understanding of its present situation and modifies the team's approach to dealing with those issues. The non-sequential models do not have a predetermined sequence of events. Rather, most of the models can be viewed as “contingency models” because the observed development patterns are largely the result of internal and external factors of the team such as time. Within the non-sequential models, also hybrid models and process models could be grouped, as they do not propose a specific pattern of team development. Although Smith (2001) did not further subdivide the development models into more specific subcategories, it is possible to do so for the linear progressive and non-sequential models (see Table 1 ).

### 2.2 Development patterns

Smith's (2001) categorization of team development models ( Table 1 ) shows that a variety of (self-managing) team development patterns can be found in the literature. When considering SMTs in particular, it can be suggested that the non-sequential models of team development are, in general, most representative for permanent naturally occurring teams in organizations. Most of them represent teams as open rather than closed systems by indicating that teams not only find ways of dealing with internal group problems, but also with the context within the organization in which they are embedded and the context outside of that organizations' boundaries ( Smith, 2001 ). Next to this, empirical evidence often deviates from the linear and cyclical sequential models of team development. Instead, permanent naturally occurring SMTs were found to move through the phases in a different order or developed in ways that cannot be described by these stages ( Wellins *et al.* , 1991 ; De Leede and Stoker, 1996 ; Kuipers and De Witte, 2005 ; Kuipers and Stoker, 2009 ). Taking the analysis on the non-sequential models a step further, it seems that the process models of Dunphy and Bryant (1996) , Kuipers and Stoker (2009) and Powell and Pazos (2017) are most suitable for describing SMTs' development pattern. Process models allow for the consideration of various development processes as simultaneous, which makes this perspective more dynamic than the clearly demarcated phases of the time-based and hybrid non-sequential models ( Kuipers and Stoker, 2009 ). Nevertheless, the perspective on SMT development will not rely solely on the process models as it can be argued that the different types of non-sequential models can be viewed as being complementary. The non-sequential models of Kuipers and Stoker (2009) , Kozlowski *et al.* (1999) , Salas *et al.* (2005) and Peralta *et al.* (2018) merely attempted to describe the patterns of (self-managing) team development. By doing this, these models have neglected the internal and external factors that affect the development pattern of (self-managing) teams. In contrast, the remaining non-sequential models of Dunphy and Bryant (1996) , Gersick (1988) , Mcgrath (1991) , Morgan *et al.* (1993) and Powell and Pazos (2017) highlight the importance of such factors. Hence, different types of non-sequential models contribute to our understanding of team development: Kuipers and Stoker (2009) , Kozlowski *et al.* (1999) , Salas *et al.* (2005) and Peralta *et al.* (2018) are preoccupied with describing in what way (self-managing) teams develop, while Dunphy and Bryant (1996) , Gersick (1988) , Mcgrath (1991) , Morgan *et al.* (1993) and Powell and Pazos (2017) are focusing on factors that cause changes in the way teams develop. Therefore, in our perspective on SMT development, the various non-sequential models are integrated by arguing that SMTs develop toward effective functioning along a series of processes that may follow one another in a variety of possible patterns or may take place simultaneously over time. Importantly, each of these processes is influenced by internal and external factors of the SMT, which are described in section 2.4 .

### 2.3 Defining aspects of development processes

Although a variety of team development patterns can be found in the literature, a more in-depth content analysis of the non-sequential models, linear-progressive and cyclical models shows that they all exhibit strong similarities on 13 key aspects (see Table 2 ), which cover all fundamental insights of the team development literature. An adoption and revision of Kuipers and Stokers' (2009) processes for describing SMT development is used for grouping these key aspects into the following three processes: team management, task management and boundary management and improvement. Team management refers to the interpersonal feelings and behaviors among team members, while the task management involves “the extent to which the team manages its primary process” ( Kuipers and Stoker, 2009 , p. 408). The third category, boundary management and improvement, represents the extent to which the team handles external relations and engages in activities of continuous improvement and advanced management and support. The difference between our development processes and those of Kuipers and Stokers (2009) can be found in the process of “team management.” Two additional key aspects identified in the literature were added to this process, including “backup behavior” and “motivating and confidence building.” In addition, this study has attempted to create a more general overview since Kuipers and Stokers' (2009) processes are characterized by context-specific terms, for example, “initiating and supporting product and process improvements” and “customer and supplier relations.” In Table 2 , the three development processes and its 13 underlying key aspects are described and linked to those authors who explicitly referred to it.

### 2.4 Factors

The next and final step to arrive at a perspective on SMT development concerns the explicit identification of the factors that influence the development pattern of SMT's. As shown in Table 3 , the eight factors found across the literature can be organized by the individual-, team-, organizational- or environmental level. At the individual level, two factors were found to positively influence SMT development: individual human capital (i.e. the level of team members' skill and learning abilities) and positive team member attitudes toward working in SMTs. Prior studies with regard to individual human capital suggest that SMT development lasts longer or is even inhibited when initial skill levels and learning abilities are low ( Van Amelsvoort and Benders, 1996 ; Dunphy and Bryant, 1996 ), especially with respect to task management and boundary management and improvement ( Balkema and Molleman, 1999 ). Some studies also mentioned that team member attitudes may enhance or seriously restrict SMT development ( Van Amelsvoort and Benders, 1996 ; Hut and Molleman, 1998 ; Balkema and Molleman, 1999 ), due to different individual psychological needs and motives: while some like challenging jobs and favor the increased authority and decision-making responsibilities, do others not want to become multifunctional and prefer limited jobs ( Balkema and Molleman, 1999 ). At the team level, four factors were found: psychological safety, team turnover (i.e. team instability), team size and nature of the task. Both O' leary (2016) and Raes *et al.* (2015) mentioned that high levels of team psychological safety contribute to (self-managing) team development as it allows team members to feel comfortable enough to give their opinion, ask questions and engage in shared decision-making, which stimulates team learning processes and activities such as sharing information and solving problems. In contrast, high team turnover and team size were identified by several authors to inhibit (self-managing) team development ( Cashman *et al.* , 2004 ; Wheelan *et al.* , 2003 ; Wheelan, 2009 ). Although Mcgrath (1991) , Morgan *et al.* (1993) and Wheelan (2005) identified the team-level factor nature of the task, the specific task attributes that are important for understanding the impact of the task on (self-managing) team development are not specified by them. Factors at the organizational-level associated with SMT development are management style and material support. Studies with respect to management style addressed that managers who continue to display top-down management styles (instead of adopting the role of a facilitator and coach) not only inhibited the decentralization of authority and decision-making responsibilities to SMTs but also fostered distrust of SMT members ( Hut and Molleman, 1998 ). Some studies paid particular attention to the influence of material support in the form of training and time investments. For example, both Dunphy and Bryant (1996) and Van Amelsvoort and Benders (1996) focused on training and reasoned that such investments enable team members to obtain knowledge, skills and abilities needed for the more diverse and complicated tasks of SMTs. Finally, the environmental-level factor requisite for self-management relates to the actual need for SMTs. According to Morgan's (1986) principle of “requisite variety,” the level of self-management has to be contingent on the level of environmental variety the organization has to deal with. If, for example, the level of variety of work processes is high, work will predominantly be of a non-routine and unique nature. In such a situation, it is more difficult to find solutions in the form of standardized work processes, which makes it more desirable to allocate authority and decision-making responsibility to SMTs, i.e. a high requisite for self-management. It is for this reason that a high requisite for self-management was reported to foster SMTs in developing toward full self-regulation ( Dunphy and Bryant, 1996 ; Hut and Molleman, 1998 ; Balkema and Molleman, 1999 ).

## 3. Methodology

The perspective on SMT development was assessed in a Dutch mental healthcare organization by using a multiple method design of qualitative research methods. To this end, 14 retrospective interviews and 13 SMT observations were performed.

### 3.1 Research context

This study was performed at GGzE, a Dutch mental healthcare organization in and around the city of Eindhoven. GGzE has approximately 2.100 employees and offers ambulatory and clinical (forensic) mental healthcare to more than 16.000 registered patients (i.e. children, adolescences, adults and elderly) with complex psychiatric and psychosocial disorders in a year ( GGzE, 2017 ). The management of GGzE initiated the transition toward self-management on 1 January 2016 as a means to increase patient and employee satisfaction, reduce (redundant) administrative burden and increase treatment efficacy. Since then, a total number of 100 primary healthcare SMTs were formed, the divisional structure was reorganized into 11 units and new roles such as team advisor and process facilitator were created. To support SMTs, the self-management process facilitator provided a (self) diagnostic instrument which measured their developmental progression toward self-management. Results from this instrument ascertained that some SMTs were more developmentally advanced than others ( GGzE, 2017 ), which made GGzE an appropriate context to carry out the present study.

### 3.2 Data collection

Retrospective interviews and SMT observations were performed to gain more insight in the current practice of SMT development at GGzE. *All* 13 advisors of the 100 primary healthcare SMTs were selected for the retrospective interviews to ensure that the sample met the requirements for diversity and representation. Additionally, the self-management process facilitator was selected as the interview respondent. She had more in-depth knowledge about the overall development of the SMTs, which was a valuable contribution to the creation of a comprehensive representation. The team advisors and self-management process facilitator were interviewed face-to-face in September 2016. The retrospective interviews deployed a semi-structured format, which drew upon our perspective on SMT development. In general, interviews respondents were asked to reflect on SMTs' development pattern from January 2016 till September 2016 for each of the three defined processes and their underlying key aspects. In addition, interview respondents were asked to indicate if (and how) the identified internal and external factors influenced SMTs' development pattern and to identify any other factors that influenced these patterns. The retrospective interviews averaged 60 min in time and were audio-recorded with permission of study participants.

A number of 13 primary mental healthcare SMTs from six different units were selected for the SMT observations by following a non-probability convenience sampling strategy (see Table A1 for a detailed overview of the observed SMTs). Each SMT was observed during a feedback meeting in which SMT members discussed the results of the self-diagnostic instrument to gain insight in their developmental progression toward self-management. During the observations, the researcher acted as a non-participant and took detailed field notes of SMT members' dialogs and informal talks. The observations were conducted in the period between April 2016 and November 2016. The observations averaged one hour in time and were overt, meaning that all SMT members were informed about the study and knew why the observer was there. To the best of our knowledge, no bias was created by the non-participant SMT observations.

### 3.3 Data analysis

Interviews were audio-recorded and verbatim transcribed. A thematic analysis of the transcripts and observational field notes was carried out by following the three step method described by Spencer *et al.* (2013) : data management, descriptive accounts and explanatory accounts. The analysis process began with reading and rereading all collected data. The data were then coded with the software package NVivo 11. Text fragments were coded and classified into themes based on *a priori* codes of our SMT perspective and a series *a posteriori* code that emerged from the data. Coded fragments were then compared with previously defined themes to confirm and refine emerging themes. As the analysis progressed, the themes evolved and relationships were determined and mapped between themes and their respective sub-themes. After data analysis, a respondent validation exercise followed in which the themes were shared and discussed with the team advisors and self-management process facilitator during one of their regular meetings.

## 4. Results

**Figure 1:**
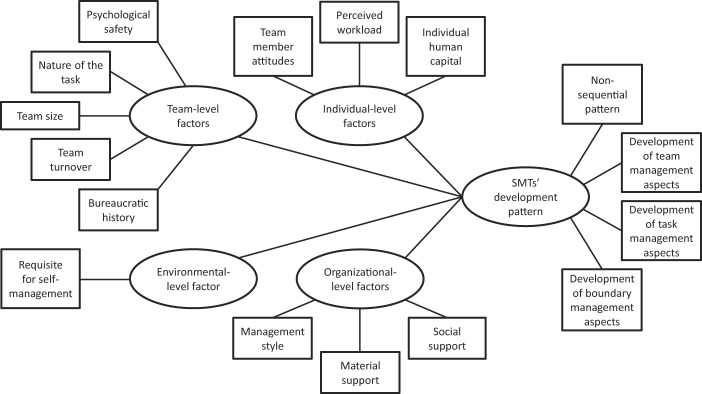
Thematic map derived from interview transcripts and observational field notes

The thematic map in Figure 1 presents the five main themes (in circles) and 16 sub-themes (in squares) developed through thematic analysis of the interview transcripts and observational field notes. Quote-illustrated results are described per theme below.

### 4.1 Development pattern of SMTs

Results of the retrospective interviews provided support for the three defined development processes of our perspective–team management, task management and boundary management and improvement–and their 13 underlying key aspects. Respondents mentioned that the perspective was comprehensive: it captured all activities teams should develop to become a fully self-managing entity. The respondent of interview 2 reflected for example the following: “ *Everything what they are doing comes really back in here. I am not missing anything.”* Next to this, results of both the retrospective interviews and SMT observations showed that SMTs did not develop along a predefined, unitary development pattern. Individual SMTs appeared to have their own strengths and weaknesses, and developed each of the three processes in a variety of possible patterns or simultaneously over time. One respondent, for example, indicated that SMTs initially concentrated on multiple key aspects, like “goal orientation”, “planning and coordination activities” and “work communication,” while others noticed that these key aspects still needed to be developed. Also the following two quotes clearly showed that SMTs developed along various patterns: *“The annual appraisals for example […] They were like: Let’s start with it, the year will be over before you know. So they do that very well”* (interview 11) and *“When it comes to annual appraisals, they all find that still very difficult and complicated. For this year we have decided to do that with the managers”* (interview 12).

With regard to each of the three development processes separately, several results of both the retrospective interviews and SMT observations are worth to mention. First, results showed that SMTs could further develop the process team management to two key aspects that remained behind in all SMTs, namely “conflict management” and “mutual performance monitoring and backup behavior.” Interview respondents explained this by the low sense of psychological safety, making it more difficult to disclose concerns, seek feedback and/or ask for help. Results of the retrospective interviews and SMT observation concerning the second process, task management, emphasized that also the key aspect “performance management” remained behind in all SMTs. One reason is that three other key aspects, i.e. “goal orientation,” “mutual performance motoring and backup behavior,” and “decision-making and control” needed to be resolved first, before SMTs were able to monitor and review progression toward goals (thereby providing some support for the linear-progressive models of team development). The respondent of interview 13 explained this as following: “ *They are not sufficiently developed to manage their performance. To do this, it is very important that you stick to agreements, provide feedback to each other, and expect significant changes. These steps must all precede.* ” Other reasons that were mentioned by interview respondents and SMT members to prevent SMTs from developing “performance management” are: deficit individual human capital, high-perceived workload and limited information sources (see next paragraph). Finally, retrospective interviews and SMT observations indicated that the overall development of the third process, boundary management and improvement, was highly dispersed: some SMTs were already highly developmentally advanced in “external relations” and “advanced tasks,” while others perceived these activities as “ *a bridge too far* ” (interview 10).

### 4.2 Factors

The retrospective interviews and SMT observations showed that the various development patterns were largely the result of the factors of our perspective on SMT development, with the exemption of requisite for self-management. Next to these, three additional factors were found to influence SMTs' development processes in practice as well, namely: perceived workload, bureaucratic history and social support. An overview of these factors is presented by Table 3 .

#### Individual level

First, results of the retrospective interviews and SMT observations showed that individual human capital and positive team member attitudes positively influence the development of all three development process. However, individual skills and qualifications largely varied within multidisciplinary SMTs, which restricted the development of the key aspect “multi-functionality.” With regard to team members' attitudes, most members were found to be highly motivated to carry out tasks related to the primary process, whereas they perceived regulatory tasks like “performance management” and “advanced management and support” as particularly disturbing and time-consuming. The respondent of interview 1 mentioned for example: “ *We just want to do where we have been hired for. That is working with patients. I hear that very often. They just see it [self-management] as an extra workload.”* Finally, a factor that appeared to constrain SMT development is high-perceived workload. Both the retrospective interviews and SMT observations revealed that SMT members had to cope with heavy workloads and could therefore not find the time for several key aspects. One SMT member illustrated this as follows: “ *I think that we are working really hard. We just keep going without thinking”* (observation 8).

#### Team level

On the team level, five factors were found to affect the development pattern of SMTs. The first factor, psychological safety, was indicated to allow SMT members to feel comfortable enough to engage with various key aspects of the three development processes. Nonetheless, most interview respondents stressed that the overall sense of psychological safety within SMTs was low: SMT members were often reluctant to discuss problems, ask critical question, and provide each other with feedback because they believed that any of this would be perceived as destructive rather than constructive, as mentioned in interview 5: *“Everybody wants to remain at the club, so they hardly dare to be critical. That is something I have noticed, a small amount of feedback has been given.”* Second, interview respondents and SMT members stated that the development of team management and task management can be seriously stagnated or even deteriorated by high team turnover. The reason is that SMTs with high team turnover needed to continuously redevelop each of the three processes, which made it extremely difficult to achieve progression. At the same time, it was also emphasized by interview respondents that new SMT members could offer alternative ideas and viewpoints, indicating that the overall influence of team turnover on SMTs' development processes may not be negative, but rather inverted U-shaped. A similar effect was found for team size. Both results of the retrospective interviews and SMT observations showed that large SMTs encountered more difficulties with developing key aspects such as “goal orientation,” “work communication” and “decision-making and control,” while too small SMTs had insufficient capacity to perform and develop such key aspects. Results with regard to the fourth factor, nature of the task, revealed that two tasks attributes have a substantial influence on SMTs' development processes. The first task attribute, type of healthcare service, distinguished care on ambulatory and clinical basis. These types of healthcare services were found to be particularly influential on the development of boundary management and improvement as interview respondents mentioned that SMS' in ambulatory care were much more developmentally advanced in managing “external relations” than SMTs in clinical care. This difference was explained by the fact that SMTs in the clinical care needed to have continuous contact with their patients, whereas SMTs in the ambulatory care needed to work with entire communities, including family members, municipalities and chain partners. Furthermore, some interview respondents mentioned that the type of healthcare service also influenced the development of the process team management. Primary tasks in ambulatory care were indicated to be more individualistic in nature and demand less continuity, which ensured that SMTs in ambulatory care could more easily develop “planning and coordination activities.” The respondent of interview 13 reflected this by saying: “ *That is a totally different way of working. It makes a great difference for example with scheduling or creating a duty list together. Everyone just makes their own schedules and appointments in ambulatory care, while you just have to make sure that there is 24 h a day a workforce available in clinical care. This requires more effort from an organizational point of view.”* The second task attribute, patient population, related to the severity and complexity of the psychiatric or psychosocial disorder of SMTs' patients. Interview respondents stated that the most severe and complex patient population not only needed long-term, specialist treatment, but was also involved in the criminal justice system. As the tasks of SMTs providing care to this patient population were substantially more complex (i.e. confirming to strict guidelines, be constantly aware of team members and patients' safety, not being able to afford any mistake), interview respondents observed that these SMTs were less developmentally advanced and required a certain degree of supervision. The fifth and final identified team level factor is SMTs' bureaucratic history. Several interview respondents emphasized that the management style of SMTs' prior manager influenced how easily SMTs developed. SMTs that were previously directed by a severe top-down management style were, in comparison with SMTs that were previously directed by a facilitating management style, observed to move less quick through each of the three development processes because members of such SMTs were not used or even afraid to perform the higher share of responsibilities associated with self-management. One respondent illustrated this as following: *“If you are really used to a manager who did everything for you, it would have given you less food for thought. But when an organization asks: Hey, start thinking on your own. Yeah, that is a major shock.”* (interview 3).

#### Organizational level

Next to the factors at the individual and team level, some factors at the organizational level were found to be important as well. A factor that was mentioned explicitly by interview respondents and SMTs is management style. Interview respondents stressed that it is essential that managers provide room for self-management. However, most of them were indicated to find it difficult to let go control and trust SMTs in making the right decisions, especially when the financial results were disappointing or when SMTs had to deal with a considerable number of serious untoward incidents. As a result, management was found to inhibit or decline the decentralization of several key aspects to SMTs. The respondent of interview 10 mentioned the following about the style of a manager: *“One team, for example SMT X, actually said to their manager: Give it all to us, we want to do it, give us that responsibility. […] However, their manager said: No, I do not trust it, I want to control it. If you are sick, you call me. And your annual appraisals? Start doing core business first.”* With regard to the factor material support, the results revealed, next to training and time investments, another form that is critical for SMT development, namely information sources. First, lack of time investments were found to be a major obstacle for all three development processes. According to interview respondents, management undertook too many organizational changes at once, making SMT members feel pressured and hindering them in getting familiar with different key aspects. Training investments were also deemed important as it ensured that SMT members learned to work effectively together and obtained diverse skills to be able to perform the higher share of responsibilities. The second form, information sources, was found to be necessary for SMTs to be able to manage itself and, hence, to develop all three development processes. Finally, results of the retrospective interviews revealed that social support by the team advisor played a crucial role in fostering all three development processes. They not only helped SMTs by giving information and asking reflective questions but also paid attention to SMT member development by individually coaching them. Moreover, team advisors were found to act as “linking pins” by providing connections between SMTs and passing relevant information from the SMT to higher-level management and vice versa. Similar findings were found for members of the support staff such as HR, finance and information and communications technology (ICT). They were indicated to be particularly valuable for developing key aspects of the processes of task management and boundary management and improvement as they could offer social support and transfer their own experience to SMTs.

#### Environmental level

Because a highly complex and dynamic environment was found for *all* SMTs, no specific influence of requisite for self-management on SMTs' development processes could be detected. Interestingly, however, results of the retrospective interviews showed that that the high requisite for self-management hindered SMTs in their development as it was too much to effectively regulate; SMT members were observed to be often confused, not to know how to take appropriate action, or even to paralyze when confronted with changes in their environment. The following quote reflected this clearly: *“I wonder whether it has a positive impact on the level of self-management. […] If teams are well-advanced in their process and something suddenly changes, they have to adjust their entire process again. Teams often find this very difficult. […] Sometimes they really start panicking.”* (interview 2).

## 5. Discussion

**Figure 2:**
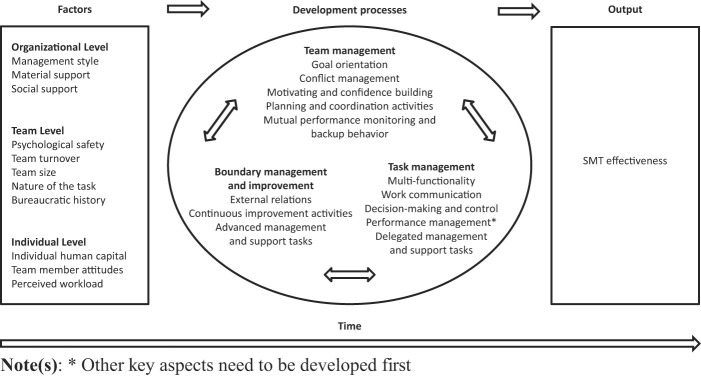
Non-sequential model of SMT development

The present study contributes to the body of knowledge on team development by examining the development of SMT's in healthcare. Based on an exploration of different team development models and identification of related key aspects and factors that were found across the models, a perspective on SMT development was presented. Whereas linear-progressive and cyclical models specifically state that team development does take place in predetermined phases or stages, our perspective suggested that SMTs develop along a non-sequential pattern of three processes–team management, task management and boundary management and improvement–that is largely the result of several individual, team, organizational and environmental-level factors. Empirical results confirmed the proposed perspective on SMT development and provided additional support for the non-sequential models in literature (except for the key aspect “performance management”). SMTs were found to reach effective functioning by developing each of the three defined processes in a variety of possible patterns or simultaneously over time, depending on many factors of the perspective on SMT development. Study results also revealed three additional factors that influence SMTs' development pattern as well, including perceived workload, bureaucratic history and social support. By combining and structuring the insights of the theoretical exploration and empirical analysis, it is possible to create a non-sequential SMT development model based on McGrath's (1964) input-process-output (I-P-O) model of teams (see Figure 2 ). The I-P-O model is an open system approach and posits, in line with this study, that a variety of inputs (factors) influence processes (development processes), which in turn contribute to team outputs (SMT effectiveness). Although our model suggests that phase theories are not suitable for describing SMTs' development pattern, it is not meant to question them. Results of this study demonstrated that SMT's are open systems where the observed patterns of development are largely the result of intragroup factors and the context in which they are embedded. Since linear- progressive and cyclical models rarely accommodated such influences, it seems that these models are still very helpful for proposing development patterns of self-analytic groups (e.g. therapy or laboratory groups) where contingencies are often absent or strictly controlled. The various development models are therefore not incompatible, but need to be viewed as explaining similar phenomena from different perspectives ( Chidambaram and Bostrom, 1997 ).

The findings of this study also suggest two other implications that contribute to current research and theory on the development and implementation of SMTs. First, by revealing crucial factors for SMT development at the individual, team and organizational levels, it is suggested by this study that SMT development requires a thorough consideration of SMTs' internal and external context (i.e. a holistic approach). Mainstream literature on SMT development often ignores contextual influences and promotes the idea that it is just a matter of restructuring the organization and delegating responsibilities to teams ( Wellins *et al.* , 1991 ; Van Amelsvoort and Benders, 1996 ). Study results add to this line of thinking and argue that the concept of organizational culture is also significant for understanding SMT development. It directs the values and behaviors of organizations' members and determines the existence of some of the revealed factors such as team member attitudes toward working in SMT's and management style. In this regard, merely treating SMT implementation as a relatively simple structural task without considering the change of the traditional top-down culture that challenges and treats autonomy and responsibly of all organizations' members in itself is not enough. Rather, to foster SMT development, it is suggested that cultural change need to be secured alongside with structural change to direct the values and behaviors of organizations' members in a manner compatible with the decentralized SMT structure. This is congruent with a warning in the organizational change literature that many change initiatives often fail to materialize as planned without considering the aspect of organizational culture ( Alvesson and Sveningsson, 2015 ). However, as argued by Cameron and Quinn (2011) , the problem with changing organizational culture is that it is very difficult to achieve, not only because culture is implicit in nature and difficult to identify, but also because once set values and behaviors are difficult to modify.

A second implication of this study concerns the feasibility of SMTs in healthcare. Although most studies reflect the common belief that use of SMTs generally improves organizational effectives, healthcare organizations may not necessarily benefit from them. Results of this study demonstrate that the feasibility and development of SMTs is constrained by some specific features of its context, including the environment, the nature of the task, the organizational culture and individual human capital. The first feature relates to the environmental level factor requisite for self-management. In contrast with the literature ( Dunphy and Bryant, 1996 ; Hut and Molleman, 1998 ; Balkema and Molleman, 1999 ), study results revealed that SMTs' highly complex and dynamic environment did not favor SMT development as it was too much to effectively deal with. This might indicate, as pointed out by the paradox of flexibility ( Volberda, 1996 ), that healthcare organizations not only must implement SMTs to be able to adapt quickly to dynamic environments but also need a context of stability to avoid chaos and stimulate SMT development. Second, in terms of nature of task, the work processes of care for patients with severe and complex needs are also suggested to limit the feasibility for self-management. In this study, SMTs providing specialized long-term forensic psychiatric care were found to require a certain degree of supervision because of their substantially more complex tasks (i.e. confirming to strict guidelines, be constantly aware of team members and patients' safety, not being able to afford any mistake). Similar findings were found in the literature: in medical trauma teams, it was observed that when a patient was more severely injured, decisions must be made quickly with limited time for thorough discussion, thereby favoring a directive instead of an empowering leadership style ( Ford *et al.* , 2016 ). Next, the present study indicates that also an attribute of the third feature, organizational culture, may constrain SMT development in healthcare. Results show that the overall low sense of psychological safety in the team context inhibited SMT development, especially with respected to “conflict management” and “mutual performance monitoring and backup behavior”. According to Edmondson (2004) , the overall low sense of psychological safety found in this study can be attributed to the culture in healthcare. She explained that healthcare's emphasis on individual vigilance encourages professionals to take responsibility to solve problems as they arise. This, however, creates barriers to psychological safety and, in turn, SMT development because it is considered as a weakness to ask help and rude to bother other busy team members to let them know something has gone wrong ( Edmondson, 2004 ). Finally, results regarding individual human capital of SMT members demonstrate that there are practical limits for developing the key aspect “multi-functionality” to support job enlargement and increase flexibility. This with respect to multidisciplinary mental healthcare SMTs since specialized skills and sharp institutional work rule restrictions, like licensing requirements, were found to prevent such SMT's from becoming effectively multiskilled. This finding complements past research of Dunphy and Bryant (1996) , who have stated that the competency levels required for some tasks may require specific and intense investment of training that may exceed the estimated value of the flexibility to be achieved. The development of “multi-functionality” seems therefore only feasible for monodisciplinary healthcare SMTs or tasks that involve low skill complexity.

Although the above implications emphasize that SMT development appears to be more difficult than the literature has told us, it does not mean that one should simply disregard the concept of self-management in healthcare. Some implications might be overcome or could be deployed in combination with the bureaucratic principles for organizations, as suggested by the practice implications in the next section.

### 5.1 Limitations and future research

Despite its contributions, this study has some limitations that offer relevant directions for future research. First, an important methodological limitation of this study concerns the retrospective nature of the interviews. Memory recall issues of the study participants and the way in which they filtered memories based on current beliefs may limit the accuracy and completeness of the data of the retrospective interviews. A real-time process study ( Langley *et al.* , 2013 ) instead of a retrospective study is advocated to gain additional and more fine-grained insights into the development pattern of SMTs. In such longitudinal study, the period of nine months (to which this study was also limited) needs to be extended. Second, although this study shows that management and support staff are important actors in the development of SMTs, our perspective on SMT development was only assessed with SMT members and their team advisors. It would therefore be interesting to incorporate the viewpoints of management and support staff in future research to consider their supporting role more elaborate. Third, after completing the theoretical exploration in May 2016, three newly published articles in the team development literature were found ( Powell and Pazos, 2017 ; Peralta *et al.* , 2018 ; Shuffler *et al.* , 2018 ). The additional insights provided by Powell and Pazos (2017) and Shuffler *et al.* (2018) regarding three of the identified factors, nature of the task, material support and social support, could therefore not be taken into account in the theoretical exploration. Fourth, this study was conducted within a mental healthcare organization with clinical and ambulatory SMTs. Future research should involve other care delivery settings as well to verify whether the results of the retrospective interviews and SMT observations align with these cases and create a more general approach to SMT development in healthcare. Finally, another opportunity for future research is to look at the relationship between the three development processes and SMT effectiveness to ensure that SMT development in healthcare is not a goal in itself, but a means to achieve certain desired organizational goals ( Kuipers and Stoker, 2009 ). In this respect, the contribution provided by Dunphy and Bryant (1996) and Kuipers and Stoker (2009) might offer a useful starting point as they made a connection between SMT development and the effectiveness outcomes of performance and quality of work life.

## 6. Practical implications

The findings of this study provide several important practical implications for SMT development in healthcare organizations. A first, rather general advice for managers, advisors and policymakers in healthcare is that they should not depend on linear-progressive and cyclical phase models to stimulate SMT development as they might overlook elements that are worthwhile to consider. Rather, they are encouraged to continuously monitor the development of SMTs through the lens of our non-sequential model to identify key aspects and factors that are open for improvement. Such approach of continuous evaluation and improvement should be regarded from the process perspective of organizational change, a more dynamic and emergent approach than the theories of planned change ( Alvesson and Sveningsson, 2015 ). Furthermore, the findings of this study indicate that SMT development requires a major change in both the structure and culture of the entire organization. Since the cultural change process starts with disconfirmation, which may cause denial and resistance to change ( Schein, 2010 ), it is suggested to make team members feel psychologically safe to ensure that they are comfortable enough to engage with the higher share of responsibilities associated with self-management and have positive attitudes toward it. Edmonson (2004) recommended that managers can support psychological safety by exemplifying the desired behavior. Practically, they could for instance be accessible and open to questions, ask questions themselves, admit mistakes, demonstrate criticism and engage in the behavior of giving and asking for feedback. Moreover, to prevent that managers may undermine the decentralization of authority and decision-making responsibilities to SMTs by top-down management styles, particular attention could be devoted to their role. To give SMTs time to get familiar with self-management, it is recommended that managers alter their traditional role of a supervisor gradually into the role of a facilitator, whereby, over time, an increased number of key aspects are handed down to the SMT. However, one should not assume that all SMTs can deal with the same amount of authority and decision-making responsibilities over time. For each SMT, it is suggested to consider the nature of the task (in terms of type of healthcare service and patient population) and, based on this, decide if one opt “total” self-management or less far-reaching forms of self-management. The abovementioned implications are recommended to not solely be the task of managers, advisors and policymakers. Active involvement of team members is also necessary in order to create room for self-management.

## Figures and Tables

**Table 1:** Categorization of team development models

Linear-progressive models	Cyclical models	Non-sequential models
*Group dynamics models*	Marks *et al.* (2001)	*Time-based models*
Tuckman (1965) Tuckman and Jensen (1977)	Bushe and Coetzer (2007)	Gersick (1988) Mcgrath (1991)
Wheelan (2005)		*Hybrid models*
*Socio-technical phase models*		Kozlowski *et al.* (1999)
Van Amelsvoort and Benders (1996) Hut and Molleman (1998) *Consultancy models* Wellins *et al.* (1991)		Morgan *et al.* (1993) Salas *et al.* (2005) Peralta *et al.* (2018) *Process models*
		Dynphy and Bryant (1996) Kuipers and Stoker (2009) Powell and Pazos (2017)

**Table 2:** Key aspects of team development models

Key aspect	Description	Authors
*Team management*		
Goal orientation	Identification and prioritization of goals and sub-goals	Tuckman (1965) , Wellins *et al.* (1991) , Mcgrath (1991) , Kozlowski *et al.* (1999) , Marks *et al.* (2001) , Wheelan (2005) , Bushe and Coetzer (2007) , Kuipers and Stoker (2009) , Powell and Pazos (2017) , Peralta *et al.* (2018)
Planning and coordinating activities	Planning of work and support activities and orchestrating the sequence and timing of these activities.This includes identifying team members' capabilities and specifying team members' roles	Tuckman (1965) , Wellins *et al.* (1991) , Mcgrath (1991) , Morgan *et al.* (1993) , Dunphy and Bryant (1996) , Van Amelsvoort and Benders (1996) , Hut and Molleman (1998) , Kozlowski *et al.* (1999) , Marks *et al.* (2001) , Wheelan (2005) , Bushe and Coetzer (2007) , Kuipers and Stoker (2009) , Powell and Pazos (2017)
Conflict management	Working through task and interpersonal disagreements among team members	Tuckman (1965) , Mcgrath (1991) , Morgan *et al.* (1993) , Marks *et al.* (2001) , Wheelan (2005) , Bushe and Coetzer (2007) , Kuipers and Stoker (2009) , Peralta *et al.* (2018)
Mutual performance monitoring and backup behavior	Assisting team members to perform their tasks by providing verbal feedback or coaching, helping behaviorally in carrying out actions, or assuming and completing a task	Wellins *et al.* (1991) , Hut and Molleman (1998) , Kozlowski *et al.* (1999) , Marks *et al.* (2001) , Salas *et al.* (2005) , Kuipers and Stoker (2009) , Powell and Pazos (2017)
Motivating and confidence building	Generating and preserving a sense of collective confidence, motivation, and task-based cohesion with regard to task accomplishment	Hut and Molleman (1998) , Marks *et al.* (2001) , Kuipers and Stoker (2009) , Peralta *et al.* (2018)
*Task management*		
Multi-functionality	Developing multi-functionality to support job enlargement	Wellins *et al.* (1991) , Dunphy and Bryant (1996) , Van Amelsvoort and Benders (1996) , Hut and Molleman (1998) , Kuipers and Stoker (2009)
Work communication	Exchanging task related information	Wellins *et al.* (1991) , Van Amelsvoort and Benders (1996) , Wheelan (2005) , Kuipers and Stoker (2009) , Powell and Pazos (2017) , Peralta *et al.* (2018)
Decision-making and control	Joint performance of managerial tasks	Wellins *et al.* (1991) , Mcgrath (1991) , Dunphy and Bryant (1996) , Van Amelsvoort and Benders (1996) , Hut and Molleman (1998) , Bushe and Coetzer (2007) , Kuipers and Stoker (2009) , Peralta *et al.* (2018)
Delegated management and support tasks	Carrying out and arranging routine support activities (e.g. plan and organize team meetings)	Wellins *et al.* (1991) , Dunphy and Bryant (1996) , Van Amelsvoort and Benders (1996) , Hut and Molleman (1998) , Kuipers and Stoker (2009)
Performance management	Tracking task and progress toward goal accomplishment and transmitting progress to team members in order to increase team	Wellins *et al.* (1991) , Morgan *et al.* (1993) , Dunphy and Bryant (1996) , Van Amelsvoort and Benders (1996) , Hut and Molleman (1998) , Marks *et al.* (2001) , Kuipers and Stoker (2009) , Powell and Pazos (2017)
*Boundary management and improvement*		
Continuous improvement activities	Identifying opportunities and developing plans for improvement and innovation	Wellins *et al.* (1991) , Morgan *et al.* (1993) , Dunphy and Bryant (1996) , Van Amelsvoort and Benders (1996) , Hut and Molleman (1998) , Kozlowski *et al.* (1999) , Marks *et al.* (2001) , Salas *et al.* (2005) , Kuipers and Stoker (2009) , Powell and Pazos (2017) , Peralta *et al.* (2018)
External relations	Handling of relations with other teams or individuals who provide inputs or receive outputs from the team	Wellins *et al.* (1991) , Dunphy and Bryant (1996) , Van Amelsvoort and Benders (1996) , Hut and Molleman (1998) , Bushe and Coetzer (2007) , Kuipers and Stoker (2009)
Advanced management and support activities	Carrying out and arranging non-routine support activities (e.g. personnel selection, annual appraisal)	Wellins *et al.* (1991) , Dunphy and Bryant (1996) , Van Amelsvoort and Benders (1996) , Hut and Molleman (1998) , Kuipers and Stoker (2009)

**Source(s)** : Adapted and revised from Kuipers and Stoker, 2009

**Table 3:** Factors that influence SMTs' development pattern

Theoretical exploration					Empirical analysis	
Factor	Influence			Authors	Influence	Data source(s)
*Individual level*						
Individual human capital	+			Dunphy and Bryant (1996) , Van Amelsvoort and Benders (1996) , Balkema and Molleman (1999) , Powell and Pazos (2017)	+	Retrospective interviews
Team member attitudes	±			Van Amelsvoort and Benders (1996) , Hut and Molleman (1998) , Balkema and Molleman (1999)	±	Retrospective interviews, SMT observations
Perceived workload	Not described				−	Retrospective interviews, SMT observations
*Team level*						
Psychological safety	+			Raes *et al.* (2015) , O' leary (2016)	+	Retrospective interviews, SMT observations
Team turnover	−			Wellins *et al.* (1991) , Cashman *et al.* (2004) , O' leary (2016)	∩	Retrospective interviews, SMT observations
Team size Nature of the task Bureaucratic history	− Not described Not described			Hut and Molleman (1998) , Wheelan *et al.* (2003) , Wheelan (2009) , Powell and Pazos (2017) Mcgrath (1991) , Morgan *et al.* (1993) , Wheelan (2005)	∩ ± ±	Retrospective interviews, SMT observations Retrospective interviews Retrospective interviews
*Organizational level*						
Management style	±			Hut and Molleman (1998) , Balkema and Molleman (1999)	±	Retrospective interviews, SMT observations
Material support	+			Wellins *et al.* (1991) , Morgan *et al.* (1993) , Dunphy and Bryant (1996) , Van Amelsvoort and Benders (1996) , Cashman *et al.* (2004)	+	Retrospective interviews, SMT observations
Social support	Not described				+	Retrospective interviews
*Environmental level*						
Requisite for self- management		+	Dunphy and Bryant (1996) , Hut and Molleman (1998) , Balkema and Molleman (1999)		Not identified	Retrospective interviews

**Note(s)** : + = Positive influence, − = Negative influence, ± = Both positive and negative influence, ∩ = Inverted *U* -shaped influence

**Table A1:** Overview of observed SMTs

No	Type of healthcare service	Number of SMT members present	Instrument score*
1	Intensive clinical treatment (closed ward) for multi-morbid complex mental health problems	5	1.8
2	Clinical treatment (closed ward) for psychotic disorders	14	3.0
3	Ambulatory treatment for multiple mental healthcare problems	8	2.3
4	Daycare and activities	8	2.5
5	Daycare and activities	6	3.0
6	Forensic mental healthcare	6	3.0
7	Daycare and activities	8	2.2
8	Clinical and ambulatory treatment for autism disorders	21	2.0
9	Ambulatory treatment for multiple mental healthcare problems	12	1.6
10	Clinical treatment (closed ward) for psychotic disorders	10	2.8
11	Neuropsychiatric treatment	7	1.7
12	Clinical treatment for autism disorders	16	2.6
13	Clinical treatment (closed ward) for psychotic disorders	15	2.1

**Note(s)** : *(Self) diagnostic instrument based on the phase model of Van Amelsvoort and Benders (1996)

Scores are ranging from 1 (phase 1) to 4 (phase 4)

## Appendix

Table A1
